# Bilateral Asymmetry in Knee and Hip Musculoskeletal Loading During Stair Ascending/Descending in Individuals with Unilateral Mild-to-Moderate Medial Knee Osteoarthritis

**DOI:** 10.1007/s10439-023-03289-9

**Published:** 2023-07-23

**Authors:** Sirui Liu, Pouya Amiri, Alison H. McGregor, Anthony M. J. Bull

**Affiliations:** 1 Department of Bioengineering, Imperial College London, Sir Michael Uren Hub, Imperial College London White City Campus, 86 Wood Ln, London, W12 0BZ UK; 2 Department of Surgery and Cancer, Imperial College London, Sir Michael Uren Hub, Imperial College London White City Campus, 86 Wood Ln, London, W12 0BZ UK

**Keywords:** Knee osteoarthritis, Musculoskeletal model, Joint loading, Stair activities, Quadriceps inhibition, Compensation strategy

## Abstract

Most cases of unilateral knee osteoarthritis (OA) progress to bilateral OA within 10 years. Biomechanical asymmetries have been implicated in contralateral OA development; however, gait analysis alone does not consistently detect asymmetries in OA patient gait. Stair ambulation is a more demanding activity that may be more suited to reveal between-leg asymmetries in OA patients. The objective of this study was to investigate the between-leg biomechanical differences in patients with unilateral mild-to-moderate knee OA. Sixteen unilateral mild-to-moderate medial knee OA patients and 16 healthy individuals underwent kinematic and kinetic analysis of stair ascent and descent. Stair ascent produced higher loading and muscle forces in the unaffected limb compared to the OA limb, and stair descent produced lower loading on the OA limb compared to healthy subjects. These biomechanical differences were apparent in the ankle, knee, and hip joints. The implications of these findings are that OA patients rely more heavily on their unaffected sides than the affected side in stair ascent, a strategy that may be detrimental to the unaffected joint health. The reduction in affected limb loading in stair descent is thought to be related to minimizing pain.

## Introduction

Knee osteoarthritis (OA) is highly prevalent, associated with an aging population, and causes pain and mobility impairments. OA instigation and progression are associated with activities that induce high mechanical loading within the joint [39], such as stair ascending and descending, squatting, kneeling, and long-time standing [22, 23, 35, 38, 42].

The most commonly studied activity to investigate the mechanical environment of the knee in OA is gait, where knee adduction moment (KAM) is used as a surrogate of articular contact forces within the tibiofemoral joint; KAM reflects medial compartment joint contact forces [40], predicts structural progression of knee OA [8, 28, 51], and reflects varying degrees of knee OA severity [3]. However, KAM is not always correlated with medial tibiofemoral contact forces and does not necessarily provide a correct understanding of the articular tibiofemoral loading during gait [59]. Meanwhile, the use of gait on its own provides a partial measure only of overall joint load during activities of daily living. When compared to gait, stair ascending and descending produce higher knee joint loading [41], and stair ambulation is reported to be the first activity that causes pain in OA patients. Therefore, this activity has been proposed as a marker for early detection of OA [29]. As such, assessing articular loading in stair ascent and descent in knee OA patients will enable the development of strategies to delay disease progression.

Compared to healthy individuals, knee OA patients have lower knee flexion angle and ankle dorsiflexion angle in both stair ascent and descent [26, 30], and in stair ascending they present with greater hip flexion/trunk forward lean angles [2, 26]. Such kinematic adaptations result in lower knee flexion moment (KFM) [24, 36] that might be a compensatory strategy to reduce the burden on quadriceps muscles in knee OA patients.

80% of patients with unilateral knee OA develop OA on their contralateral knee over a period of 5 to 12 years [48]. This leads to the hypothesis that biomechanical symmetry is relevant to OA. Studies on symmetry in gait have been inconsistent with some showing kinematic asymmetries in unilateral [14] and bilateral OA [47], with others reporting no differences in unilateral OA [50]. In addition, an understanding of possible asymmetries in muscle and joint contact forces between the affected and unaffected side of OA patients is lacking. Quantifying these differences at the level of muscle and contact forces potentially provides biomarkers for early detection of the disease and offers targets (e.g., muscle force reduction) for interventions to delay or halt the disease progression; the more demanding tasks of stair ambulation may provide more detailed information regarding the existing asymmetries in unilateral knee OA.

Musculoskeletal modeling can quantify articular contact forces within the tibiofemoral joint as well as muscle activation strategies by using inverse dynamics calculations and muscle optimization methods [17]. These have been validated for tibiofemoral contact forces using instrumented knee prostheses [25], hip contact forces [1], and for muscle activation using electromyography [16, 44]. Therefore, it is appropriate to use musculoskeletal modeling to assess compartmental loading of the tibiofemoral joint in stair ambulation.

The objective of this study was to investigate the biomechanical and musculoskeletal loading asymmetries between affected and unaffected legs of unilateral mild-to-moderate knee OA patients and healthy individuals during the demanding task of stair ascending/descending. We hypothesized that unilateral knee OA patients would develop movement strategies as well as muscle activation strategies to reduce loading on their affected side, thereby increasing the loading on their unaffected leg, a compensation method that is detrimental to joint health and can increase the possibility of generating bilateral knee OA in the long term.

## Materials and Methods

### Participants

Sixteen individuals (9 females and 7 males) with mild-to-moderate unilateral medial knee OA participated in this study (Table 2); the participants’ experimental data were taken from a large OA dataset [18, 19]. The patients were diagnosed with unilateral medial knee OA according to their Kellgren–Lawrence score and clinical and physical examination by an experienced clinician, where a KL score of 1 and 2–3 were classified as having mild and moderate OA, respectively [37]. The other leg did not show any symptomatic presence of knee OA. Sixteen healthy individuals, matched with the OA group for gender, height, age (±5 years), with no clinical evidence of knee OA or any other neuromuscular disease also participated in the study (Table 2). All data collection process had ethical approval from the South West London Research Ethics Committee, and all participants provided written informed consent.

### Stair Ascending and Descending Experimental Data Collection

All participants walked up/down stairs; for stair ascending, they performed at least three trials, starting and ending with the right foot strike (a whole gait cycle) and at least another three trials starting and ending with the other (left) foot strike. For stair descending, similarly at least three trials for each side were performed. Thus, at least 12 trials were performed by each participant. The step depth and tread height were 255 mm and 170 mm, respectively.

Twenty reflective markers were placed on specific bony landmarks of both legs (8 on each leg) and the pelvis (4 markers), and 4 marker clusters were placed on participants’ shanks and thighs (Table 1). The 3D positions of the markers were captured by a 10 camera Vicon motion capture system (Vicon Motion Systems Ltd, Oxford, UK) during the stair activities with a sampling rate of 100 Hz. In addition, synchronous bilateral ground reaction forces (GRF) of the participants were obtained with a sampling rate of 1000 Hz using two Kistler force platforms (Kistler Type 9286B, Kistler Instrument AG, Winterthur, Switzerland), which were embedded separately into the two steps of the stairs [18, 19].

**Table 1:** Optical motion tracking marker locations

Marker	Location
RASIS	Right anterior superior iliac spine
LASIS	Left anterior superior iliac spine
RPSIS	Right posterior superior iliac spine
LPSIS	Left posterior superior iliac spine
C1, C2, C3	Additional markers placed on calf
T1, T2, T3	Additional markers placed on thigh
FM2	Head of the second metatarsal
FCC	Calcaneus
FMT	Tuberosity of the fifth metatarsal
TF	Additional marker placed on the foot
FAM	Apex of the lateral malleolus
TAM	Apex of the medial malleolus
FLE	Lateral femoral epicondyle
FME	Medial femoral epicondyle

### Musculoskeletal Model

FreeBody, a three-dimensional segment-based musculoskeletal modeling software, was used to determine kinematics, kinetics, and muscle and joint contact forces [12] during stair ascending activities for both legs of all participants during a whole gait cycle (defined as one heel strike to the next heel strike of the same foot). FreeBody has been validated for the prediction of knee contact forces during different activities using instrumented knee implant data [16].

A fourth-order Butterworth low-pass filter with a cut-off frequency of 4 Hz was used to filter marker position data; synchronous GRF data were also down-sampled and filtered with the same filter. Subsequently, the processed maker data from a static trial and the marker and GRF data from the dynamic trials were used as input to Freebody to perform inverse kinematics, inverse dynamics, and musculoskeletal modeling calculations.

The FreeBody model consists of 5 segments, including foot, shank, patella, thigh, and pelvis, and 4 joints, ankle, tibiofemoral, patellofemoral, and hip joints. FreeBody uses quaternion algebra to determine the kinematics of a segment movement (rather than joints), allowing 6 degrees of freedom (DOF) for the segment [10, 11]; the prediction of the in-vivo knee contact forces from an instrumented implant demonstrated that constraining the relative movement of thigh to pelvis (i.e., hip joint with only 3 rotational DOF) improved the predictions; thus this has been constrained in this study [16]. The kinetics of the movement, i.e., net moments and joint forces, are calculated using a wrench formulation by Dumas et al. [20].

To estimate muscle, ligament, and articular contact forces, FreeBody performs a static optimization, minimizing the sum of cubed muscle activations, constrained by the equations of motion of the musculoskeletal systems and maximum force of the muscles, as demonstrated in Eq. (1) [17]:

1 $$ \begin{gathered} \min J = \mathop \sum \limits_{i = 1}^{163} \left( {\frac{{F_{i} }}{{F_{i\max } }}} \right)^{3} \hfill \\ subject \,to: \hfill \\ 0 \le F_{i} \le F_{i\max } i = 1, \ldots ,163 \hfill \\ \left[ {\begin{array}{*{20}c} {\mathop \sum \limits_{l = 1}^{L} F_{l} \cdot {\varvec{n}}_{{{\varvec{lm}}}} - \mathop \sum \limits_{k = 1}^{K} F_{k} \cdot {\varvec{n}}_{{{\varvec{k}}\left( {{\varvec{m}} - 1} \right)}} + {\varvec{J}}_{{\varvec{m}}} - {\varvec{J}}_{{{\varvec{m}} - 1}} } \\ {\mathop \sum \limits_{l = 1}^{L} F_{l} \cdot {\varvec{n}}_{{{\varvec{lm}}}} \times {\varvec{r}}_{{{\varvec{lm}}}} - \mathop \sum \limits_{k = 1}^{K} F_{k} \cdot {\varvec{n}}_{{{\varvec{k}}\left( {{\varvec{m}} - 1} \right)}} \times {\varvec{r}}_{{{\varvec{k}}\left( {{\varvec{m}} - 1} \right)}} - \widetilde{{{\varvec{d}}_{{\varvec{m}}} }} \times {\varvec{J}}_{{{\varvec{m}} - 1}} } \\ \end{array} } \right] \hfill \\ = \left[ {\begin{array}{*{20}c} {M_{m} {\varvec{E}}_{{{\mathbf{3 \times 3}}}} } & {{\mathbf{0}}_{{{\mathbf{3 \times 3}}}} } \\ {M_{m} \widetilde{{{\varvec{c}}_{{\varvec{m}}} }}} & {{\varvec{I}}_{{\varvec{m}}} } \\ \end{array} } \right]\left[ {\begin{array}{*{20}c} {{\varvec{a}}_{{\varvec{m}}} - g} \\ {\varvec{\ddot{\theta }}_{{\varvec{m}}} } \\ \end{array} } \right] + \left[ {\begin{array}{*{20}c} {{\mathbf{0}}_{{{\mathbf{3 \times 1}}}} } \\ {\dot{\varvec{\theta }}_{{\varvec{m}}} \times I_{m} \dot{\varvec{\theta }}_{{\varvec{m}}} } \\ \end{array} } \right] \hfill \\ \end{gathered}, $$

where ${F}_{i}$ is force of the ${i}_{th}$ muscle elements (*i* = 1,…,163), and ${F}_{i{\text{max}}}$ represents maximum force of the ${i}_{th}$ muscle elements. $m$ represents the segment number (numbering from distal to proximal), $L,K$ represent the number of proximal and distal muscle element at ${m}_{th}$ segment, ${{{\varvec{n}}}_{{\varvec{m}}},{\varvec{n}}}_{{\varvec{m}}-1}$ represent the line of action of the proximal and distal muscle element, ${{{\varvec{r}}}_{{\varvec{m}}},{\varvec{r}}}_{{\varvec{m}}-1}$ represent the moment arm of the proximal and distal muscle element, ${{{\varvec{J}}}_{{\varvec{m}}},{\varvec{J}}}_{{\varvec{m}}-1}$ represent the proximal and distal joint reaction force, ${I}_{m}$ is the inertia tensor, ${\ddot{{\varvec{\theta}}}}_{{\varvec{m}}},{\dot{{\varvec{\theta}}}}_{{\varvec{m}}}$ represent the angular acceleration and angular velocity, $g$ is the gravitational acceleration, ${M}_{m}$ is the ${m}_{th}$ segment mass, ${{\varvec{E}}}_{3\times 3}$ is the identity matrix. ${{\varvec{c}}}_{{\varvec{m}}},{{\varvec{d}}}_{{\varvec{m}}}$ are the vector from the proximal joint to the segment COM and from the proximal to the distal joint, and $\widetilde{{c}_{m}}$ and $,\widetilde{{d}_{m}}$ are the skew symmetric matrix of ${c}_{m}$ and ${d}_{m}$. The equations of motion are posed for the segments, resulting in a total of 22 equations: 18 equations describing the linear and angular motions of foot, shank, and thigh; 3 equations describing linear movement of patella, and an equation accounting for force balance between quadriceps muscles and patellar ligament [20].

To determine muscle and joint geometry for each subject (required for the equations of motion), an anatomical Atlas containing the musculoskeletal datasets of 10 subjects with different dimensions was used [17]. Each anatomical dataset contains the geometry, including origin, insertion, and via points of 163 muscle elements (representing 38 lower limb muscles). The dataset also contains bone geometries, joints centers of rotation, and wrapping surfaces of the curved lines-of-action of iliopsoas and gastrocnemius. The tibiofemoral joint is compartmentalized into medial and lateral components through two contact points on the femoral condyles. The maximum force of each muscle is obtained by multiplying its physiological cross-sectional area by an assumed fixed value for the maximum muscle stress equal to 31.39 N/cm [3, 20].

To obtain the musculoskeletal geometry for each subject, a musculoskeletal dataset from the anatomical Atlas was selected and scaled to match the dimension of the subject. It has been shown that predicted muscle and joint contact forces using a scaled musculoskeletal model are accurate if a musculoskeletal anatomical dataset with the same gender and similar anthropometric data is used [9, 15, 17]. The anatomical dataset that minimized the error in the prediction of knee contact forces was selected using Eq. (2) [17].

2 $$ RMSD =\;5.32 + \Delta LL + 0.38 \times \Delta Mass + 37.22 \times \Delta Ratio + 7.75\Delta {\text{gender}}, $$

where $RMSD$ is the estimated root mean square deviation between predicted and measured tibiofemoral contact forces, $\Delta LL$ is the difference in lower limb length of the subject of interest and the subject from the anatomical Atlas, $\Delta Mass$ is the difference in mass, and $\Delta Ratio$ is the difference in lower limb length-to-pelvis width (RASIS to LASIS distance) ratio; $\Delta gender$ is 0 if a model with the same gender is used, otherwise 1. To scale the selected anatomical dataset for each subject the muscle attachment sites, joint rotation centers, and wrapping surfaces in the original anatomical dataset were multiplied by a scaling factor as shown in Eq. (3) [17]:

3 $$  \begin{gathered}   \left( {x,y,z} \right) = sf \times \left( {x_{0} ,y_{0} ,z_{0} } \right) \hfill \\   sf_{{{\text{Pelvis}}}}  = \frac{{{\text{Subject}}\,{\text{pelvis}}{\mkern 1mu} \,{\text{width}}}}{{{\text{Anatomical}}{\mkern 1mu} \,{\text{model}}{\mkern 1mu} \,{\text{pelvis}}{\mkern 1mu} \,{\text{width}}}} \hfill \\   sf_{{{\text{Thigh}}}}  = \frac{{{\text{Subject}}{\mkern 1mu} \,{\text{thigh}}\,{\mkern 1mu} {\text{length}}}}{{{\text{Anatomical}}{\mkern 1mu} \,{\text{model}}{\mkern 1mu} \,{\text{thigh}}{\mkern 1mu} \,{\text{length}}}} \hfill \\   sf_{{{\text{Shank}}}}  = \frac{{{\text{Subject}}\,{\mkern 1mu} {\text{shank}}\,{\mkern 1mu} {\text{length}}}}{{{\text{Anatomical}}{\mkern 1mu} \,{\text{model}}\,{\mkern 1mu} {\text{shank}}\,{\mkern 1mu} {\text{length}}}} \hfill \\   sf_{{{\text{Foot}}}}  = \frac{{{\text{Subject}}{\mkern 1mu} \,{\text{foot}}{\mkern 1mu} \,{\text{length}}}}{{{\text{Anatomical}}{\mkern 1mu} \,{\text{model}}{\mkern 1mu} \,{\text{foot}}\,{\mkern 1mu} {\text{length}}}}, \hfill \\  \end{gathered}   $$

where the $sf$ is the segment scaling factors ($seg$ can be foot, shank, thigh, and pelvis).$({x}_{0},{y}_{0},{z}_{0})$ is the muscle point coordinates in the segment coordinate system of the anatomical model, and $(x,y,z)$ is the resulting muscle point coordinates in the segment coordinate system of the scaled model.

### Data Analysis and Statistical Testing

Several important features of the obtained waveforms from inverse kinematics, inverse dynamics, and musculoskeletal modeling were extracted for further statistical analysis in both stair ascending and descending for both sides of all subjects. These were peak knee flexion angle, peak hip adduction angle, 1st and 2nd peaks of hip flexion angles, 1st and 2nd peaks of ankle inversion and dorsiflexion moments, 1st and 2nd peaks of the KAM, KAM impulse, 1st and 2nd peaks of KFM, 1st and 2nd peak of hip adduction moment, 1st and 2nd peaks and impulses of total medial compartment knee contact force as well as its separate components: compressive, anterior shear, medial shear, and 1st and 2nd peaks of total hip contact force. Finally, peak forces of major muscles around the knee and hip joints were also estimated in this study, comparing vastus medialis (VM), vastus lateralis (VL), vastus intermedius (VI), rectus femoris (RF), medial gastrocnemius (MG), lateral gastrocnemius (LG), long head of biceps femoris (BFLH), short head of biceps femoris (BFSH), semimembranosus (SM), semitendinosus (ST), gluteus medius (GMED), gluteus maximum (GMAX), iliacus (IL), and psoas major (PSOAS). All intersegmental moments were normalized by subject mass and forces were normalized by their body weight. These data were processed as follows: for each key parameter for the knee OA group, 3 values for 3 trials were obtained for each side and the mean values computed. Walking speed was also obtained using the position of FM2 markers during the gait cycle. Outliers were discarded for each parameter before any statistical analysis (Eq. (4)):

4 $$ \begin{gathered} B_{\max } = q3 + \left( {1.5 \times \left( {q3 - q1} \right)} \right) \hfill \\ B_{\min } = q1 - \left( {1.5 \times \left( {q3 - q1} \right)} \right) \hfill \\ \end{gathered}, $$

where ${B}_{max}$ and ${B}_{min}$ represent the upper and lower bounds, and $q3$ and $q1$ are the upper and lower quartiles, respectively.

Statistical analysis was carried out in Python 3.8.3. Independent Student’s t-test was used to compare the features of left and right legs of healthy individuals. The healthy group showed no significant differences between the sides, so the bilateral data were combined as one group, and generalized estimation equations [27] were used to compare OA affected leg, OA unaffected leg, and healthy (significance set at 5%). Walking speed during each stair activity was also compared between the OA and healthy group using Student’s *t* test (significance level = 0.05).

## Results

There were no statistically significant differences in age, height, mass, or BMI between the healthy and OA group (Table 2).

**Table 2:** Group participant data

Parameters	Healthy	OA	*p* values
Age (years)	55.9 ± 13.0	55.2 ± 13.0	0.94
Height (m)	1.70 ± 0.09	1.67 ± 0.09	0.48
Mass (kg)	71.3 ± 11.4	75.4 ± 11.4	0.39
BMI (kg/m^2^)	24.6 ± 2.4	26.9 ± 2.4	0.06

**Fig. 1:**
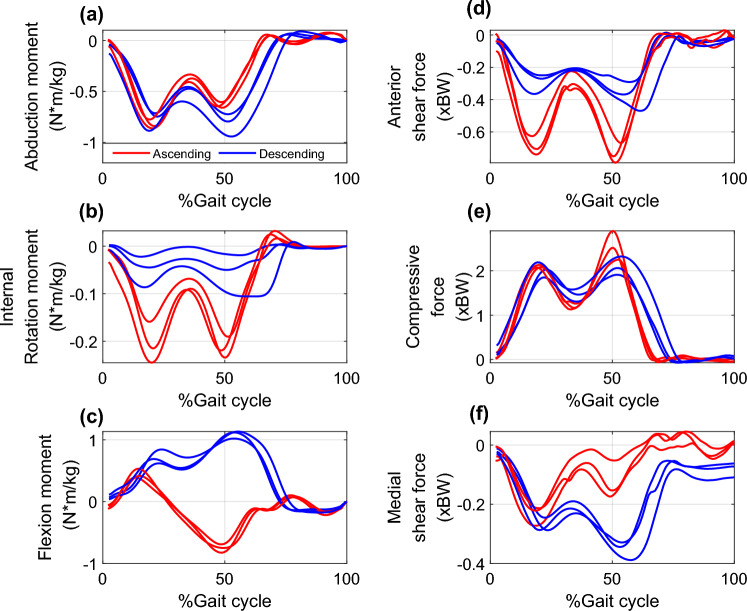
Intersegmental moments and medial compartment knee joint contact forces for a representative OA subject; **a**–**c** knee moments and **d**–**f** medial compartment knee contact forces in stair ascent and descent. Gait cycle is defined from heel strike to ipsilateral heel strike. Red lines represent three trials in stair ascending and blue lines represent three trials in the stair descending activity

**Fig. 2:**
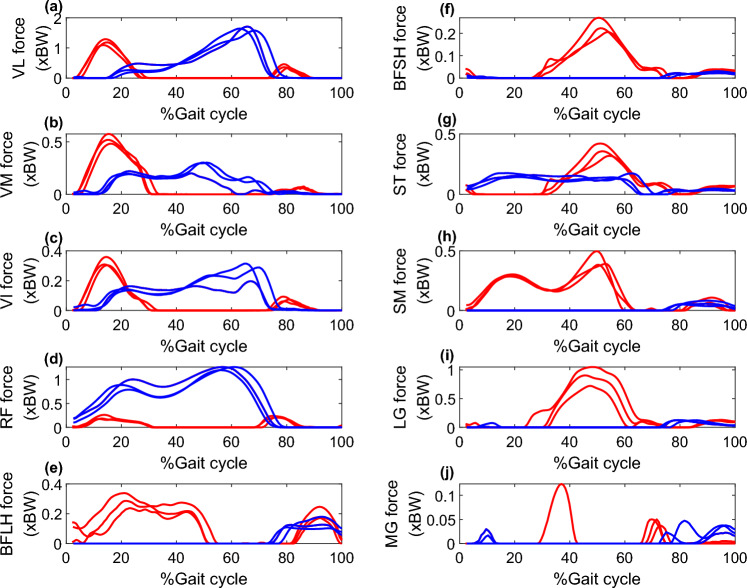
Muscle forces in stair ascending and descending for the representative OA participant in Fig. 1. Red lines represent muscle forces of three trials in stair ascending and blue lines represent muscle forces of three trials in the stair descending activity). **a**–**d** Quadriceps, **e**–**h** Hamstrings, and **i**, **j** Gastrocnemius muscle forces

Exemplar components of the medial compartment knee contact forces and intersegmental knee joint net moments, and corresponding muscle forces are shown in Figs. 1 and 2, respectively. During stair ascending, the early stance external KFM is balanced by large forces from quadriceps (vastii and RF) (Fig. 2), while in late stance the external knee extension moment is balanced by high forces from knee flexors, including hamstrings (BFSH, SM, and ST) and gastrocnemius (mostly lateral) muscles (Fig. 2). These muscle forces generate compressive loads on the medial compartment of the knee that are approximately 2 times BW. During stair descending, the sagittal plane external moment stays in flexion throughout the stance phase, requiring prolonged high forces from the quadriceps while the knee flexors show low activations (Fig. 2). This strategy is associated with peak medial compartment knee contact forces of around 2 BW and higher midstance contact forces (~ 1.5 BW).

### Stairs Ascending

**Fig. 3:**
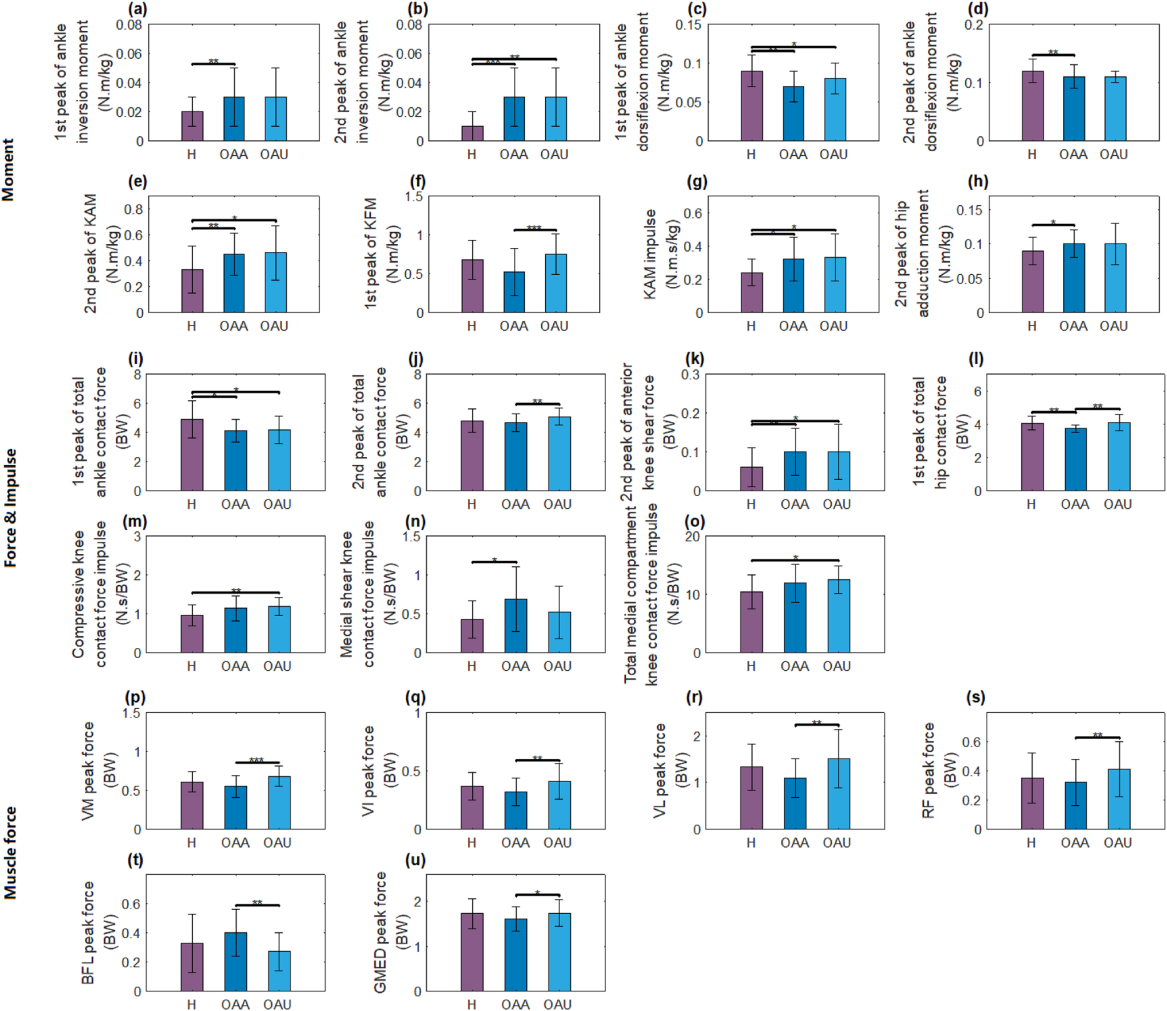
Statistically significant different force and moment features during stair ascent. (H—healthy, OAA—OA affected side, OAU—OA unaffected side). **a**–**h** Moments and moment impulse; **i**–**o** Joint force and joint force impulses; **p**–**u** Muscle peak force. *p < 0.05, **p < 0.01, ***p < 0.001

Table 3 shows the results of the statistical comparisons in stair ascent with Fig. 3 displaying those that are significantly different. The comparison results demonstrated that 1st peak of KFM, 2nd peak of total ankle contact force, 1st peak of total hip contact force, and VM, VI, VL, RF, and GMED peak forces were higher in OA unaffected vs OA affected knees, while BFL peak force was lower; 2nd peak of ankle inversion moment, 2nd peak of KAM, KAM impulse, 2nd peak of anterior shear knee contact force, compressive knee contact force impulse, and total medial compartment knee contact force impulse were higher in OA unaffected side than healthy group, whereas 1st peak of ankle dorsiflexion and 1st peak of total ankle contact force were lower. In addition, 1st peak and 2nd peak of ankle inversion moment, 2nd peak of KAM, KAM impulse, 2nd peak of hip adduction moment, 2nd peak of anterior shear knee contact force, and medial shear contact force impulse were higher in OA affected side compared to healthy, whereas 1st and 2nd peak of ankle dorsiflexion moment, 1st peak of total ankle contact force and 1st peak of total hip contact force were lower in OA affected side (Table 3). There was no difference in walking speed of healthy (0.39 ± 0.04 m/s) and OA (0.37 ± 0.05) group in stair ascending (*p* = 0.22).

**Table 3:** Waveform feature results for stair ascending (3 group analysis; mean ± standard deviation; generalized estimation equation comparison significant differences are shown in bold with differences between H and OA-affected^a^, H and OA-unaffected^b^, and OA affected and OA unaffected^c^; far right column shows overall *p* values below)

Parameters	Healthy	OA affected	OA unaffected	*p* value
Knee peak flexion angle (deg)	96.0 ± 6.6	90.5 ± 10.3	92.7 ± 8.6	(^a^0.06, ^b^0.19, ^c^0.22)
Hip peak adduction angle (deg)	− 13.2 ± 6.3	− 15.0 ± 5.2	− 16.2 ± 5.2	(0.91, 0.70, 0.38)
1st peak of hip flexion angle (deg)	− 11.2 ± 5.5	− 9.0 ± 6.4	− 10.0 ± 6.0	(0.28, 0.56, 0.35)
2nd peak of hip flexion angle (deg)	− 62.8 ± 5.8	− 62.7 ± 5.7	− 63.1 ± 7.3	(0.88, 0.96, 0.73)
1st peak of ankle inversion moment (N m/kg)	**0.02 ± 0.01**	**0.03 ± 0.02**	0.03 ± 0.02	(**0.01**^**a**^, 0.06, 0.87)
2nd peak of ankle inversion moment (N m/kg)	**0.01 ± 0.01**	**0.03 ± 0.02**	**0.03 ± 0.02**	(**0.00**^**a**^**,** **0.01**^**b**^**,** 0.91)
1st peak of ankle dorsiflexion moment (N m/kg)	**− 0.09 ± 0.02**	**− 0.07 ± 0.02**	**− 0.08 ± 0.02**	(**0.01**^**a**^**,** **0.04**^**b**^**,** 0.51)
2nd peak of ankle dorsiflexion moment (N m/kg)	**− 0.12 ± 0.02**	**− 0.11 ± 0.02**	− 0.11 ± 0.01	(**0.01**^**a**^, 0.11, 0.14)
1st peak of KAM (N m/kg)	0.56 ± 0.17	0.60 ± 0.21	0.62 ± 0.21	(0.51, 0.36, 0.80)
2nd peak of KAM (N m/kg)	**0.33 ± 0.18**	**0.45 ± 0.16**	**0.46 ± 0.21**	(**0.01**^**a**^**,** **0.02**^**b**^**,** 0.81)
1st peak of KFM (N m/kg)	0.68 ± 0.25	**0.52 ± 0.30**	**0.75 ± 0.26**	(0.08, 0.36, **0.00**^**c**^)
2nd peak of KFM (N m/kg)	− 0.37 ± 0.22	− 0.41 ± 0.21	− 0.41 ± 0.22	(0.53, 0.61, 0.90)
KAM impulse (N m s/kg)	**0.24 ± 0.08**	**0.32 ± 0.13**	**0.33 ± 0.14**	**(0.02**^**a**^**,** **0.02**^**b**^**,** 0.89**)**
1st peak of hip adduction moment (N m/kg)	0.11 ± 0.02	0.11 ± 0.02	0.11 ± 0.02	(0.84, 0.58, 0.68)
2nd peak of hip adduction moment (N m/kg)	**0.09 ± 0.02**	**0.10 ± 0.02**	0.10 ± 0.03	(**0.04**^**a**^, 0.10, 0.89)
1st peak of total ankle contact force (BW)	**4.88 ± 1.27**	**4.13 ± 0.79**	**4.16 ± 0.95**	(**0.03**^**a**^**,** **0.04**^**b**^, 0.95)
2nd peak of total ankle contact force (BW)	4.80 ± 0.79	**4.67 ± 0.59**	**5.08 ± 0.58**	(0.63, 0.28, **0.01**^**c**^)
1st peak of compressive knee contact force (BW)	1.76 ± 0.37	1.77 ± 0.37	1.75 ± 0.30	(0.89, 0.92, 0.73)
2nd peak of compressive knee contact force (BW)	1.62 ± 0.50	1.69 ± 0.47	1.75 ± 0.40	(0.70, 0.40, 0.64)
1st peak of anterior shear knee contact force (BW)	0.17 ± 0.03	0.15 ± 0.07	0.16 ± 0.05	(0.33, 0.88, 0.37)
2nd peak of anterior shear knee contact force (BW)	**0.06 ± 0.05**	**0.10 ± 0.06**	**0.10 ± 0.07**	(**0.01**^**a**^**,** **0.05**^**b**^, 0.75)
1st peak of medial shear knee contact force (BW)	0.52 ± 0.09	0.55 ± 0.16	0.52 ± 0.15	(0.49, 0.86, 0.43)
2nd peak of medial shear knee contact force (BW)	0.43 ± 0.09	0.45 ± 0.09	0.46 ± 0.10	(0.67, 0.31, 0.60)
1st peak of total medial compartment knee contact force (BW)	1.83 ± 0.38	1.85 ± 0.38	1.84 ± 0.32	(0.87, 0.91, 0.93)
2nd peak of total medial compartment knee contact force (BW)	1.65 ± 0.51	1.75 ± 0.47	1.79 ± 0.43	(0.62, 0.41, 0.74)
1st peak of total hip contact force (BW)	**4.06 ± 0.41**	**3.73 ± 0.22**	**4.08 ± 0.49**	(**0.01**^**a**^, 0.91, **0.01**^**c**^)
2nd peak of total hip contact force (BW)	3.21 ± 0.75	2.97 ± 0.33	3.23 ± 0.47	(0.16, 0.87, 0.06)
Compressive knee contact force impulse (N s/BW)	**0.96 ± 0.27**	1.14 ± 0.32	**1.19 ± 0.23**	(0.10, **0.01**^**b**^, 0.34)
Anterior shear knee contact force impulse (N s/BW)	2.99 ± 0.52	3.24 ± 0.70	2.93 ± 0.69	(0.15, 0.99, 0.10)
Medial shear knee contact force impulse (N s/BW)	**0.43 ± 0.24**	**0.69 ± 0.42**	0.52 ± 0.34	(**0.03**^**a**^, 0.19, 0.23)
Total medial compartment knee contact force impulse (N s/BW)	**10.46 ± 2.90**	11.89 ± 3.21	**12.55 ± 2.36**	(0.15, **0.02**^**b**^, 0.27)
VM peak force (BW)	0.61 ± 0.13	**0.55 ± 0.14**	**0.68 ± 0.13**	(0.18, 0.16, **0.00**)
VI peak force (BW)	0.37 ± 0.12	**0.32 ± 0.12**	**0.41 ± 0.15**	(0.20, 0.36, **0.01**^**c**^)
VL peak force (BW)	1.33 ± 0.49	**1.09 ± 0.42**	**1.51 ± 0.62**	(0.12, 0.31, **0.01**^**c**^)
RF peak force (BW)	0.35 ± 0.17	**0.32 ± 0.16**	**0.41 ± 0.19**	(0.58, 0.29, **0.01**^**c**^)
MG peak force (BW)	0.51 ± 0.37	0.40 ± 0.34	0.46 ± 0.35	(0.29, 0.63, 0.60)
LG peak force (BW)	0.64 ± 0.37	0.57 ± 0.24	0.58 ± 0.28	(0.43, 0.53, 0.89)
BFL peak force (BW)	0.33 ± 0.20	**0.40 ± 0.16**	**0.27 ± 0.13**	(0.28, 0.18, **0.01**^**c**^)
BFS peak force (BW)	0.11 ± 0.07	0.12 ± 0.06	0.10 ± 0.05	(0.56, 0.73, 0.48)
SM peak force (BW)	0.19 ± 0.07	0.21 ± 0.05	0.22 ± 0.05	(0.20, 0.14, 0.69)
ST peak force (BW)	0.18 ± 0.11	0.18 ± 0.11	0.18 ± 0.10	(0.97, 0.81, 0.85)
GMED peak force (BW)	1.73 ± 0.33	**1.61 ± 0.27**	**1.74 ± 0.30**	(0.22, 0.83, **0.02**^**c**^)
GMAX peak force (BW)	0.72 ± 0.17	0.70 ± 0.07	0.69 ± 0.14	(0.50, 0.47, 0.78)
IL peak force (BW)	0.16 ± 0.07	0.16 ± 0.03	0.16 ± 0.06	(0.86, 0.98, 0.85)
PSOAS peak force (BW)	0.16 ± 0.08	0.16 ± 0.03	0.17 ± 0.06	(0.86, 0.86, 0.97)

Groups with significant differences are given in bold

### Stairs Descending

**Fig. 4:**
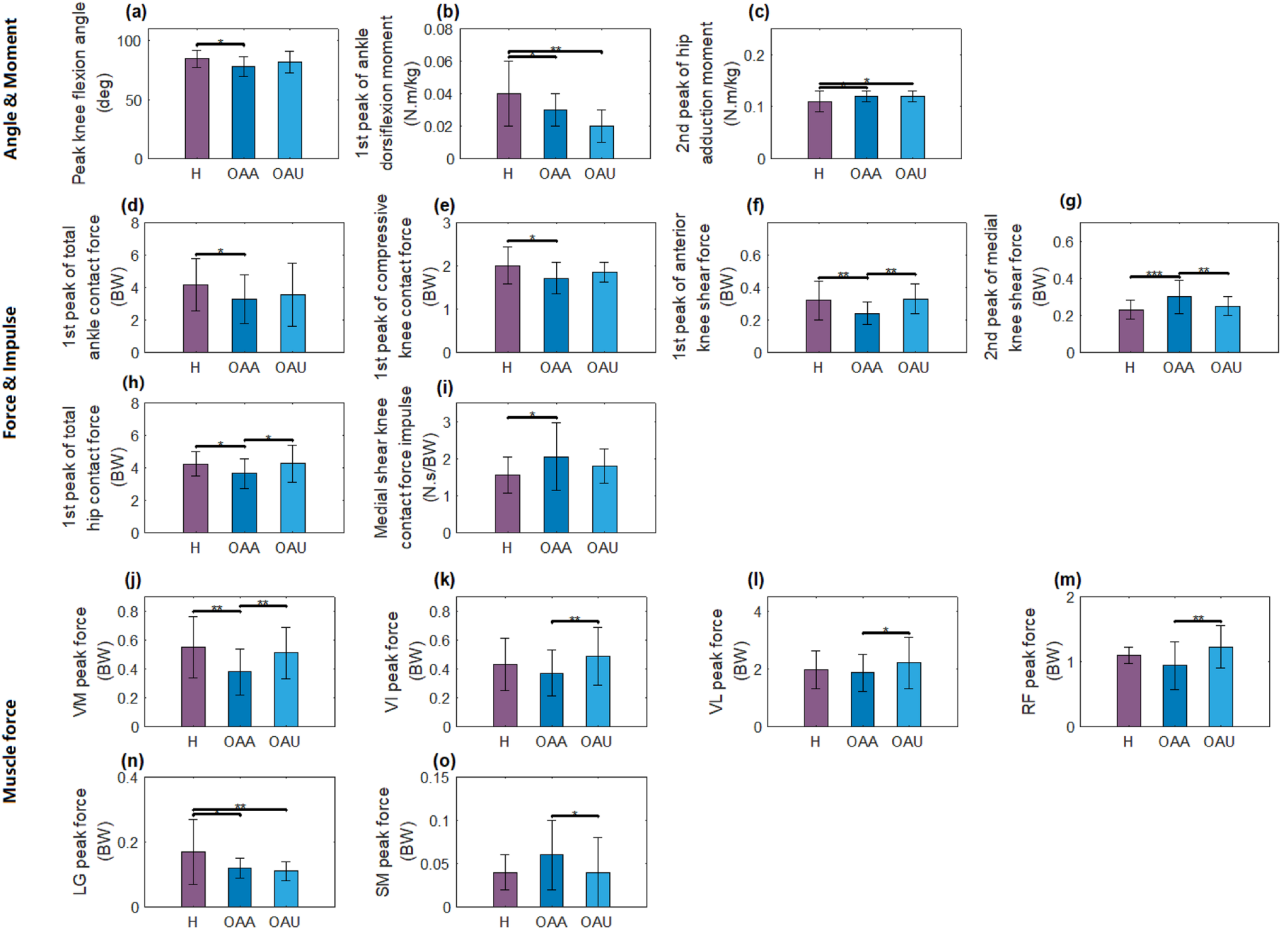
Statistically significant different force and moment features during stair descent. (H—healthy, OAA—OA affected side, OAU—OA unaffected side). **a** Angle; **b**, **c** moments; **d**–**i** joint forces and Joint force impulses; **j**–**o** muscle peak forces. *p < 0.05, **p < 0.01, ***p < 0.001

Table 4 shows the result of the statistical comparison in stair descent with Fig. 4 displaying those that are significantly different. The comparison results showed that peak knee flexion angle, 1st peak of ankle dorsiflexion moment, 1st peak of total ankle contact force, 1st peak of anterior shear knee contact force, 1st peak of compressive knee contact force, 1st peak of total hip contact force, and VM and LG muscle peak forces were higher in healthy vs OA affected groups, whereas 2nd peak of hip adduction moment, 2nd peak of medial shear knee contact force, and medial shear knee contact force impulse were lower in the healthy group. The 1st peak of ankle dorsiflexion moment and LG peak force were higher in healthy group than OA unaffected side, while 2nd peak of hip adduction moment was lower. The parameters that showed difference between the OA affected and OA unaffected side were 1st peak of anterior shear knee contact force, 1st peak of total hip contact force, VM, VI, VL, and RF peak forces, which were higher in OA unaffected side, whereas SM peak force was lower in OA unaffected side (Table 4). In addition, the healthy group descended the stairs faster than the OA group (0.62 ± 0.10 vs. 0.50 ± 0.13 m/s, *p* = 0.01).

**Table 4:** Waveform feature results for stair descending (3 group analysis; mean ± standard deviation; ANOVA significant differences are shown in bold with Tukey’s post hoc differences between H and OA-affected^a^, H and OA-unaffected^b^, and OA affected and OA unaffected^c^; far right column shows overall ANOVA p-value with pairwise p-values below)

Parameters	Healthy	OA affected	OA unaffected	*p* value
Peak knee flexion angle (deg)	**84.5 ± 7.2**	**78.0 ± 8.3**	81.7 ± 9.0	(^**a**^**0.02**, ^b^0.25, ^c^0.19)
Peak hip adduction angle (deg)	− 8.2 ± 4.3	− 9.2 ± 3.1	− 10.6 ± 4.8	(0.56, 0.15, 0.22)
1st peak of hip flexion angle (deg)	− 10.8 ± 4.9	− 8.5 ± 5.8	− 8.9 ± 5.6	(0.12, 0.37, 0.07)
2nd peak of hip flexion angle (deg)	− 34.2 ± 5.1	− 32.5 ± 5.2	− 30.8 ± 5.1	(0.20, 0.06, 0.17)
1st peak of ankle inversion moment (N m/kg)	0.01 ± 0.01	0.01 ± 0.01	0.02 ± 0.01	(0.27, 0.62, 0.19)
2nd peak of ankle inversion moment (N m/kg)	0.02 ± 0.01	0.02 ± 0.01	0.02 ± 0.01	(0.63, 0.65, 0.95)
1st peak of ankle dorsiflexion moment (N m/kg)	**− 0.04 ± 0.02**	**− 0.03 ± 0.01**	**− 0.02 ± 0.01**	(**0.02**^**a**^**,** **0.01**^**b**^, 0.25)
2nd peak of ankle dorsiflexion moment (N m/kg)	− 0.06 ± 0.02	− 0.05 ± 0.02	− 0.05 ± 0.02	(0.83, 0.71, 0.73)
1st peak of knee adduction moment (N m/kg)	0.61 ± 0.21	0.65 ± 0.18	0.66 ± 0.21	(0.57, 0.45, 0.82)
2nd peak of knee adduction moment (N m/kg)	0.69 ± 0.29	0.73 ± 0.18	0.72 ± 0.27	(0.62, 0.67, 0.99)
1st peak of KFM (N m/kg)	1.24 ± 0.25	1.11 ± 0.31	1.29 ± 0.23	(0.14, 0.56, 0.06)
2nd peak of KFM (N m/kg)	− 0.11 ± 0.02	− 0.10 ± 0.04	− 0.10 ± 0.03	(0.33, 0.18, 0.56)
Knee adduction moment impulse (N m s/kg)	0.34 ± 0.16	0.40 ± 0.13	0.42 ± 0.16	(0.21, 0.13, 0.52)
1st peak of hip adduction moment (N m/kg)	0.12 ± 0.02	0.12 ± 0.01	0.12 ± 0.02	(0.68, 0.97, 0.66)
2nd peak of hip adduction moment	**0.11 ± 0.02**	**0.12 ± 0.01**	**0.12 ± 0.01**	(**0.03**^**a**^, **0.03**^**b**^, 0.85)
1st peak of total ankle contact force (BW)	**4.17 ± 1.61**	**3.28 ± 1.50**	3.56 ± 1.92	(**0.04**^**a**^, 0.27, 0.57)
2nd peak of total ankle contact force (BW)	4.33 ± 1.11	4.58 ± 0.97	4.22 ± 1.39	(0.45, 0.80, 0.45)
1st peak of compressive knee contact force (BW)	**2.01 ± 0.43**	**1.72 ± 0.36**	1.85 ± 0.23	(**0.02**^**a**^, 0.09, 0.29)
2nd peak of compressive knee contact force (BW)	1.81 ± 0.45	1.79 ± 0.50	1.90 ± 0.39	(0.87, 0.70, 0.50)
1st peak of anterior shear knee contact force (BW)	**0.32 ± 0.12**	**0.24 ± 0.07**	**0.33 ± 0.09**	(**0.01**^**a**^, 0.65, **0.01**^**c**^)
2nd peak of anterior shear knee contact force (BW)	0.42 ± 0.13	0.40 ± 0.16	0.43 ± 0.14	(0.85, 0.93, 0.91)
1st peak of medial shear knee contact force (BW)	0.24 ± 0.05	0.23 ± 0.06	0.24 ± 0.05	(0.35, 0.81, 0.56)
2nd peak of medial shear knee contact force (BW)	**0.23 ± 0.05**	**0.30 ± 0.09**	**0.25 ± 0.05**	(**0.00**^**a**^, 0.16, **0.01**^**c**^)
1st peak of total medial compartment knee contact force (BW)	1.98 ± 0.50	1.76 ± 0.36	1.93 ± 0.23	(0.12, 0.59, 0.16)
2nd peak of total medial compartment knee contact force (BW)	2.04 ± 0.66	1.86 ± 0.51	1.94 ± 0.41	(0.36, 0.40, 0.85)
1st peak of total hip contact force (BW)	**4.25 ± 0.77**	**3.65 ± 0.90**	**4.27 ± 1.15**	(**0.03**^**a**^, 0.93, **0.03**^**c**^)
2nd peak of total hip contact force (BW)	4.08 ± 0.62	4.09 ± 0.88	4.36 ± 1.02	(0.96, 0.43, 0.32)
Compressive knee contact force impulse (N s/BW)	1.07 ± 0.28	1.18 ± 0.29	1.24 ± 0.29	(0.25, 0.06, 0.17)
Anterior shear knee contact force impulse (N s/BW)	1.98 ± 0.85	2.32 ± 0.71	2.16 ± 0.73	(0.17, 0.53, 0.28)
Medial shear knee contact force impulse (N s/BW)	**1.55 ± 0.49**	**2.05 ± 0.91**	1.80 ± 0.46	(**0.05**^**a**^, 0.08, 0.27)
Total medial compartment knee contact force impulse (N s/BW)	11.16 ± 3.35	11.99 ± 3.07	12.64 ± 2.99	(0.32, 0.16, 0.40)
VM peak force (BW)	**0.55 ± 0.21**	**0.38 ± 0.16**	**0.51 ± 0.18**	(**0.01**^**a**^, 0.36, **0.01**^**c**^)
VI peak force (BW)	0.43 ± 0.18	**0.37 ± 0.16**	**0.49 ± 0.20**	(0.21, 0.52, **0.01**^**c**^)
VL peak force (BW)	1.98 ± 0.65	**1.87 ± 0.64**	**2.22 ± 0.89**	(0.46, 0.44, **0.03**^**c**^)
RF peak force (BW)	1.10 ± 0.13	**0.94 ± 0.37**	**1.23 ± 0.32**	(0.10, 0.21, **0.01**^**c**^)
MG peak force (BW)	0.05 ± 0.04	0.08 ± 0.07	0.05 ± 0.03	(0.31, 0.52, 0.10)
LG peak force (BW)	**0.17 ± 0.10**	**0.12 ± 0.03**	**0.11 ± 0.03**	(**0.02**^**a**^**,** **0.01**^**b**^, 0.06)
BFL peak force (BW)	0.11 ± 0.06	0.10 ± 0.06	0.08 ± 0.04	(0.65, 0.07, 0.13)
BFS peak force (BW)	0.04 ± 0.01	0.04 ± 0.01	0.04 ± 0.01	(0.37, 0.09, 0.29)
SM peak force (BW)	0.04 ± 0.02	**0.06 ± 0.04**	**0.04 ± 0.04**	(0.08, 0.62, **0.03**^**c**^)
ST peak force (BW)	0.06 ± 0.03	0.06 ± 0.03	0.06 ± 0.03	(0.89, 0.62, 0.57)
GMED peak force (BW)	1.68 ± 0.39	1.75 ± 0.21	1.84 ± 0.48	(0.43, 0.26, 0.33)
GMAX peak force (BW)	0.42 ± 0.12	0.36 ± 0.13	0.40 ± 0.13	(0.20, 0.75, 0.23)
IL peak force (BW)	0.21 ± 0.08	0.16 ± 0.06	0.20 ± 0.08	(0.06, 0.67, 0.13)
PSOAS peak force (BW)	0.22 ± 0.08	0.17 ± 0.06	0.20 ± 0.09	(0.06, 0.63, 0.14)

Groups with significant differences are given in bold

## Discussion

This study has quantified kinematics, kinetics, and musculoskeletal loading asymmetries between affected and non-affected legs of unilateral mild-to-moderate knee OA patients in stair ambulation and compared this to a healthy matched control group (Table 5 summarizes all the significant differences with a large effect size, i.e., effect size ≥ 0.8) [7]. During stair ascent, the OA patients generated higher VM and VL muscle forces (and lower BFL) to counteract a larger external KFM on their unaffected side (compared to their OA side), a strategy that was associated with 24% higher compressive contact force impulse on their unaffected sides compared to healthy individuals (this was the case, even though KAM impulse was higher in both sides of OA patients compared to healthy subjects, Table 5). In addition, the OA patients had altered the loading on their unaffected hip and ankle joints: they generated higher contact forces on their unaffected vs. affected hip joints, while they loaded both affected and unaffected ankles with lower dorsiflexion and higher inversion moments compared to the healthy group. These findings show that during stair ascent, patients with unilateral mild-to-moderate knee OA climb the stairs by relying more on their unaffected knee and hip joints, while they load both ankles similarly, but differently to the healthy group. These strategies can be detrimental to contralateral knee and hip joint health and potentially leading to the development of bilateral OA.

**Table 5:** Summary of the significant differences in stair ambulation

Parameter	Groups	Effect size
2nd peak of ankle inversion moment	OAA > H OAU > H	1.3 1.3
1st peak of ankle dorsiflexion moment	H > OAA H > OAU	1.6 1.2
Knee		
1st peak KFM	OAU > OAA	0.8
KAM impulse	OAU > H OAA > H	0.8 0.8
Compressive knee contact force impulse	OAU > H	0.9
Medial shear knee contact force impulse	OAA > H	0.8
VM peak force	OAU > OAA	1.0
VL peak force	OAU > OAA	0.8
BFL peak force	OAA > OAU	0.9
1st peak of total hip contact force	H > OAA OAU > OAA	1.0 0.8
*Descent*		
Ankle		
1st peak of ankle dorsiflexion moment	H > OAU	1.0
Knee		
Knee flexion angle	H > OAA	0.8
1st peak of compressive knee contact force	H > OAA	0.8
1st peak of anterior shear knee contact force	H > OAA OAU > OAA	0.8 1.1
2nd peak of medial shear knee contact force	OAA > H	1.0
VM peak force	H > OAA OAU > OAA	0.9 0.8
RF peak force	OAU > OAA	0.8
LG peak force	H > OAA H > OAU	0.8 0.9
Hip		
1st peak of hip contact force	H > OAA	0.8

OAA, OAU, and H represent OA affected, OA unaffected, and healthy, respectively.

The effect size was calculated using Cohen’s *D*. < and > show which group had higher values for each parameter.

During stair descent, the OA patients descended the stairs slower than the healthy group and reduced their tibiofemoral compressive and anterior shear contact forces (by 16.9% and 33.3%, respectively) and quadriceps and gastrocnemius muscle forces (by 44.7% for VM and 41.6% for LG) on their OA side compared to healthy subjects (Table 5); the only difference between the unaffected side and OA side was the RF and VM muscle forces, in which the unaffected side had higher values (by 17% for RF and 34% for VM), as well as anterior shear knee contact force. These findings show that during stair descent OA patients seem to reduce the loading on their OA side at both knee and hip by slower walking, possibly to reduce pain, a strategy that may be beneficial in reducing the disease progression. Overall, the findings of this study suggest that it is essential to explore the sources of these walking asymmetries, for example, poor muscle activation or muscle weakness, and then develop interventions and strategies to target improving such underlying defects in order to enhance bilateral symmetry, to help avoid development of bilateral knee OA in patients with unilateral mild-to-moderate knee OA.

We found that knee flexion angle of OA patients’ affected sides was smaller than that of the healthy group during stair descending. During stair ascent, the knee flexion angle was not statistically smaller on the OA knee, but the difference was close to significant (*p* = 0.08, Table 3). Reduced knee flexion angle in both stair activities has been consistently reported in the previous studies and generally considered a method to avoid joint pain by lowering the maximum joint bending [33].

Large KAM has been reported in patients with knee OA during walking [3, 32, 53], however, there is no sufficient evidence for higher KAM during stair ascent/descent of OA patients (compared to healthy individuals) [34, 36, 55, 58]. Our results showed significantly higher KAM impulse in both affected and non-affected sides of OA patients compared to healthy individuals during stair ascent, with no difference between limbs. The compressive contact forces in our study, however, (obtained using a validated musculoskeletal model) [16] were more sensitive in detecting differences between the groups. In stair ascent, the impulse of the compressive contact force was higher on the unaffected knee of OA patients compared to the healthy subjects. Such high compressive forces have been implicated in pathomechanics of knee OA and can lead to cartilage degeneration and OA development in the unaffected side [49]. These higher contact forces were associated with significantly higher VM and VL forces in the unaffected knees, which counteracted the higher 1st peak of KFM in the unaffected sides. This provides support for the importance of KFM in assessing the internal environment of the joint, as it has been recently demonstrated that KAM and KFM both are required to predict the medial tibiofemoral contact forces [44, 46]. Our study would support this.

In stair descent, the affected knee showed lower peak compressive and anterior but higher medial shear contact forces (all on the medial compartment) than the healthy individuals. Lower compressive forces during stair descending on the OA side seems like a strategy to reduce loading of the joint to avoid pain [34] and may be a desired strategy to slow the progression of the disease. These lower contact forces are in agreement with smaller peak VM and RF on the affected knee compared to healthy, since quadriceps are the main contributors to 1st peak of compressive knee contact forces [60]. Smaller quadriceps forces could have been due to lower quadriceps muscle strength in patients with knee OA, suggested to be a risk factor for development and progression of the disease [54, 56]. Atherogenic quadriceps muscle inhibition, a central mechanism to reduce muscle activation could also result in lower muscle forces. It was in fact reported that quadriceps muscles were inhibited and 40% weaker than the intact side in individuals with unilateral early-stage knee OA [31]. Strength exercises have been shown to improve both muscle strength and neural drive and consequently improve function and joint pain [4, 45]. Functional electrical stimulation as an adjunct to physiotherapy is an additional effective quadriceps muscle strengthening technique for the rehabilitation older people with knee OA who have trouble with voluntary exercise [6, 21], suggesting that this could be used as a strategy to improve muscle recruitment more generally in OA patients. We also observed from the video footage that almost all OA patients had difficulty in stair descent activities, and they chose to go down the stairs sideways, reflected by an initial sideways rotation of the whole limb from the pelvis. This strategy may in fact have led to higher medial shear force in patients’ affected knees and could contribute to the cartilage wear [43] and suggests that a suitable clinical intervention could be to focus on a frontal stair descent.

Our results showed that loading at the hip and ankle joints was also associated with the knee OA on one side. The OA patients had lower early stance (1st peak) hip contact force in their affected than healthy subjects in both stair ascending and descending, while they loaded their unaffected side’s hip joint more than the affected side in stair descent. Although the hip contact forces have not been previously examined before in the presence of knee OA, in agreement with our results, it has been reported that knee OA patients walk with lower hip joint loading, in particular lower hip adduction moment, in normal gait [3, 13, 52]. The smaller hip adduction moment in OA patients has been linked to the weaker hip abductors, in particular gluteus medius and minimus, whose smaller forces result in pelvis drop and larger hip adduction angle. The smaller hip contact forces that were found here during stair ambulation were probably associated with the weaker hip abductors, which are the main contributors to the hip contact forces [9]. Although the stronger hip abductor has been suggested to stabilize upper body and consequently contribute to smaller KAM, strengthening hip abductors in patients with knee OA has not reduced the KAM and only improved the symptoms [5, 57]. The role of hip muscular loading and its relation to knee OA pathomechanics are still unclear and warrants further investigation, particularly utilizing tools that enable medial knee joint reaction force to be quantified.

In addition, the OA patients adopted the same loading on both unaffected and affected ankles, with lower dorsiflexion and higher inversion moments than healthy subjects. In agreement with these results, it has been previously shown that OA patients tend to walk with lower ankle dorsiflexion [3]; the moment reduced progressively from healthy to moderate-to-severe knee OA patients. Therefore, it seems such incremental changes in ankle moment changes could be considered a biomarker, showing the initiation/progression of the disease at the unaffected knee of unilateral OA patients.

There are some limitations associated with this study. First, the musculoskeletal model used has been validated using instrumented implant and EMG data for gait, squat, and sit to stand [16]. As such data are not available for stair ascent and descent, there may be additional uncertainty in our study. Secondly, we have not explored in detail the clinical implication at this stage, which necessitates further investigation. Third, our small sample size may have resulted in not detecting some differences. We performed a post hoc power analysis for all the variables in Tables 3 and 4, which showed that the statistical power could be as low as 0.05 for some variables, which was mostly the case for variables whose mean differences (and consequently effect size) were small, thus, the differences were not important and detection of any differences required very large samples [the low power happened mostly when comparing the two OA sides, e.g., the power for the comparison of the 2nd peak of KAM between OA affected and non-affected (0.45 ± 0.16 vs. 0.46 ± 0.21) side was 0.05].

This is the first study that has comprehensively quantified bilateral asymmetries in kinematics and kinetics, and musculoskeletal loading between affected and non-affected limbs of unilateral mild-to-moderate knee OA patients and healthy individuals in both stair ascent and descent. The study found that OA patients rely on their unaffected knee to a higher extent during stair ascent, a strategy that may contribute to bilateral OA development, whereas the patients tend to unload their affected knee during stair descent, probably to avoid pain. This has implications for informing targeted exercise and rehabilitation interventions and provides a potential outcome metric to assess such interventions.

## References

[1] Amiri P, Bull MJ, Andriacchi TP (2009). Prediction of in vivo hip contact froces during common activities of daily living using a segment-based musculoskeletal model. Front. Bioeng. Biotechnol..

[2] Asay JL, Mündermann A, Andriacchi TP (2009). Adaptive patterns of movement during stair climbing in patients with knee osteoarthritis. J. Orthop. Res..

[3] Astephen JL, Deluzio KJ, Caldwell GE, Dunbar MJ (2008). Biomechanical changes at the hip, knee, and ankle joints during gait are associated with knee osteoarthritis severity. J. Orthop. Res..

[4] Baker KR, Nelson ME, Felson TD, Layne JE, Sarno R, Roubenoff R (2001). The efficacy of home based progressive strength training in older adults with knee osteoarthritis: A randomized controlled trial. J Rheumatol..

[5] Bennell KL, Hunt MA, Wrigley TV, Hunter DJ, McManus FJ, Hodges PW, Li L, Hinman RS (2010). Hip strengthening reduces symptoms but not knee load in people with medial knee osteoarthritis and varus malalignment: A randomised controlled trial. Osteoarthr. Cartil..

[6] Burch FX, Tarro JN, Greenberg JJ, Carroll WJ (2008). Evaluating the benefits of patterned stimulation in the treatment of osteoarthritis of the knee: A multi-center, randomized, single-blind, controlled study with an independent masked evaluator. Osteoarthr. Cartil..

[7] Carson, C. The effective use of effect size indices in institutional research. In 31st Annual Conference Proceedings. 41, 2012.

[8] Chehab EF, Favre J, Erhart-Hledik JC, Andriacchi TP (2014). Baseline knee adduction and flexion moments during walking are both associated with 5 year cartilage changes in patients with medial knee osteoarthritis. Osteoarthr. Cartil..

[9] Cleather DJ, Bull AMJ (2010). Lower-extremity musculoskeletal geometry affects the calculation of patellofemoral forces in vertical jumping and weightlifting. Proc. Inst. Mech. Eng. H.

[10] Cleather DJ, Bull AMJ (2012). The development of lower limb musculoskeletal models with clinical relevance is dependent upon the fidelity of the mathematical description of the lower limb. Part 2: Patient-specific geometry. Proc. Inst. Mech. Eng. H.

[11] Cleather DJ, Bull AMJ (2012). The development of lower limb musculoskeletal models with clinical relevance is dependent upon the fidelity of the mathematical description of the lower limb. Part 1: equations of motion. Proc. Inst. Mech. Eng. H.

[12] Cleather DJ, Bull AMJ (2015). The development of a segment-based musculoskeletal model of the lower limb: Introducing Freebody. R. Soc. Open Sci..

[13] Correa TA, Crossley KM, Kim HJ, Pandy MG (2010). Contributions of individual muscles to hip joint contact force in normal walking. J. Biomech..

[14] Creaby MW, Bennell KL, Hunt MA (2012). Gait differs between unilateral and bilateral knee osteoarthritis. Arch. Phys. Med. Rehabil..

[15] Ding Z, Azmi NL, Bull AMJ (2019). Validation and use of a musculoskeletal gait model to study the role of functional electrical simulation. IEEE Trans. Biomed. Eng..

[16] Ding Z, Nolte D, Tsang CK, Cleather DJ, Kedgley AE, Bull AMJ (2016). In vivo knee contact force prediction using patient-specific musculoskeletal geometry in a segment-based computational model. J. Biomech. Eng..

[17] Ding Z, Tsang C, Nolte D, Kedgley AE, Bull AMJ (2019). Improving musculoskeletal model scaling using an anatomical atlas: The importance of gender and anthropometric similarity to quantify joint reaction forces. IEEE Trans. Biomed. Eng..

[18] Duffell LD, Gulati V, Southgate DFL, McGregor AH (2013). Measuring body weight distribution during sit-to-stand in patients with early knee osteoarthritis. Gait Posture..

[19] Duffell LD, Southgate DFL, Gulati V, McGregor AH (2014). Balance and gait adaptations in patients with early knee osteoarthritis. Gait Posture..

[20] Dumas R, Aissaoui R, de Guise JA (2004). A 3D generic inverse dynamic method using wrench notation and quaternion algebra. Comput. Methods Biomech. Biomed. Eng..

[21] Durmuş D, Alayli G, Cantürk F (2007). Effects of quadriceps electrical stimulation program on clinical parameters in the patients with knee osteoarthritis. Clin. Rheumatol..

[22] Ezzat AM, Cibere J, Koehoorn M, Li LC (2013). Association between cumulative joint loading from occupational activities and knee osteoarthritis. Arthritis Care Res. (Hoboken)..

[23] Ezzat AM, Li LC (2014). Occupational physical loading tasks and knee osteoarthritis: A review of the evidence. Physiother. Can..

[24] Fok LA, Schache AG, Crossley KM, Lin YC, Pandy MG (2013). Patellofemoral joint loading during stair ambulation in people with patellofemoral osteoarthritis. Arthritis Rheum..

[25] Fregly BJ, Besier TF, Lloyd DG, Delp SL, Banks SA, Pandy MG, D’Lima DD (2012). Grand challenge competition to predict in vivo knee loads. J. Orthop. Res..

[26] Gonçalves GH, Selistre LFA, Petrella M, Mattiello SM (2017). Kinematic alterations of the lower limbs and pelvis during an ascending stairs task are associated with the degree of knee osteoarthritis severity. Knee..

[27] Hardin, J. W., and J. M. Hilbe, New York: Chapman and Hall, 277pp, 2013.

[28] Hatfield GL, Stanish WD, Hubley-Kozey CL (2015). Three-dimensional biomechanical gait characteristics at baseline are associated with progression to total knee arthroplasty. Arthritis Care Res. (Hoboken)..

[29] Hensor EMA, Dube B, Kingsbury SR, Tennant A, Conaghan PG (2015). Toward a clinical definition of early osteoarthritis: onset of patient-reported knee pain begins on stairs. Data from the osteoarthritis initiative. Arthritis Care Res. (Hoboken)..

[30] Hicks-Little CA, Peindl RD, Hubbard TJ, Scannell BP, Springer BD, Odum SM, Fehring TK, Cordova ML (2011). Lower extremity joint kinematics during stair climbing in knee osteoarthritis. Med. Sci. Sports Exerc..

[31] Hurley MV, Newham DJ (1993). The influence of arthrogenous muscle inhibition on quadriceps rehabilitation of patients with early, unilateral osteoarthritic knees. Br. J. Rheumatol..

[32] Hurwitz DE, Ryals AB, Case JP, Block JA, Andriacchi TP (2002). The knee adduction moment during gait in subjects with knee osteoarthritis is more closely correlated with static alignment than radiographic disease severity, toe out angle and pain. J. Orthop. Res..

[33] Igawa T, Katsuhira J (2014). Biomechanical analysis of stair descent in patients with knee osteoarthritis. J. Phys. Ther. Sci..

[34] Iijima H, Shimoura K, Aoyama T, Takahashi M (2018). Biomechanical characteristics of stair ambulation in patients with knee OA: A systematic review with meta-analysis toward a better definition of clinical hallmarks. Gait Posture..

[35] Jensen LK (2008). Knee osteoarthritis: Influence of work involving heavy lifting, kneeling, climbing stairs or ladders, or kneeling/squatting combined with heavy lifting. Occup. Environ. Med..

[36] Kaufman KR, Hughes C, Morrey BF, Morrey M, An KN (2001). Gait characteristics of patients with knee osteoarthritis. J. Biomech..

[37] Kellgren JH, Lawrence JS (1957). Radiological assessment of osteo-arthrosis. Ann. Rheum. Dis..

[38] Klussmann A, Gebhardt H, Nübling M, Liebers F, Perea EQ, Cordier W, Engelhardt LV, Schubert M, Dávid A, Bouillon B, Rieger MA (2010). Individual and occupational risk factors for knee osteoarthritis: Results of a case–control study in Germany. Arthritis Res. Ther..

[39] Kontio T, Viikari-Juntura E, Solovieva S (2018). To what extent do education and physical work load factors explain occupational differences in disability retirement due to knee OA? A nationwide register-based study in Finland. BMJ Open..

[40] Kumar D, Manal KT, Rudolph KS (2013). Knee joint loading during gait in healthy controls and individuals with knee osteoarthritis. Osteoarthr. Cartil..

[41] Kutzner I, Heinlein B, Graichen F, Bender A, Rohlmann A, Halder A, Beier A, Bergmann G (2010). Loading of the knee joint during activities of daily living measured in vivo in five subjects. J. Biomech..

[42] Lau EC, Cooper C, Lam D, Chan VNH, Tsang KK, Sham A, Lau EMC (2000). Factors associated with osteoarthritis of the hip and knee in Hong Kong Chinese: Obesity, joint injury, and occupational activities. Am. J. Epidemiol..

[43] Lynn SK, Kajaks T, Costigan PA (2008). The effect of internal and external foot rotation on the adduction moment and lateral-medial shear force at the knee during gait. J. Sci. Med. Sport..

[44] Manal K, Buchanan TS (2013). An electromyogram-driven musculoskeletal model of the knee to predict in vivo joint contact forces during normal and novel gait patterns. J. Biomech. Eng..

[45] McAlindon TE, Bannuru RR, Sullivan MC, Arden NK, Berenbaum F, Bierma-Zeinstra SM, Hawker GA, Henrotin Y, Hunter DJ, Kawaguchi H, Kwoh K, Lohmander S, Rannou F, Roos EM, Underwood M (2014). OARSI guidelines for the non-surgical management of knee osteoarthritis. Osteoarthr. Cartil..

[46] Meireles S, de Groote F, Reeves ND, Verschueren S, Maganaris C, Luyten F, Jonkers I (2016). Knee contact forces are not altered in early knee osteoarthritis. Gait Posture..

[47] Messier SP, Beavers DP, Herman C, Hunter DJ, DeVita P (2016). Are unilateral and bilateral knee osteoarthritis patients unique subsets of knee osteoarthritis? A biomechanical perspective. Osteoart. Cartil..

[48] Metcalfe AJ, Andersson ML, Goodfellow R, Thorstensson CA (2012). Is knee osteoarthritis a symmetrical disease? Analysis of a 12-year prospective cohort study. BMC Musculoskelet. Disord..

[49] Metcalfe AJ, Stewart C, Postans N, Dodds AL, Holt CA, Roberts AP (2013). The effect of osteoarthritis of knee on the biomechanics of other joints in the lower limbs. Bone Jt. J..

[50] Mills K, Hettinga BA, Pohl MB, Ferber R (2013). Between-limb kinematic asymmetry during gait in unilateral and bilateral mild to moderate knee osteoarthritis. Arch. Phys. Med. Rehabil..

[51] Miyazaki T, Wada M, Kawahara H, Sato M, Baba H, Shimada S (2002). Dynamic load at baseline can predict radiographic disease progression in medial compartment knee osteoarthritis. Ann. Rheum. Dis..

[52] Mündermann A, Dyrby CO, Andriacchi TP (2005). Secondary gait changes in patients with medial compartment knee osteoarthritis: Increased load at the ankle, knee, and hip during walking. Arthritis Rheum..

[53] Mündermann A, Dyrby CO, Hurwitz DE, Sharma L, Andriacchi TP (2004). Potential strategies to reduce medial compartment loading in patients with knee osteoarthritis of varying severity: Reduced walking speed. Arthritis Rheum..

[54] Øiestad BE, Juhl CB, Eitzen I, Thorlund JB (2015). Knee extensor muscle weakness is a risk factor for development of knee osteoarthritis: A systematic review and meta-analysis. Osteoarthr. Cartil..

[55] Paquette MR, Zhang S, Milner CE, Klipple G (2014). Does increasing step width alter knee biomechanics in medial compartment knee osteoarthritis patients during stair descent?. Knee..

[56] Serrão PRMS, Vasilceac FA, Gramani-Say K, Lessi GC, Oliveira AB, Reiff RBM, Mattiello-Sverzut AC, Mattiello SM (2015). Men with early degrees of knee osteoarthritis present functional and morphological impairments of the quadriceps femoris muscle. Am. J. Phys. Med. Rehabil..

[57] Sled EA, Khoja L, Deluzio KJ, Olney SJ, Culham EG (2010). Effect of a home program of hip abductor exercises on knee joint loading, strength, function, and pain in people with knee osteoarthritis: A clinical trial. Phys. Ther..

[58] Verlaan L, Boekesteijn RJ, Oomen PW, Liu WY, Peters MJM, Emans PJ, van Rhijn LW, Meijer K (2019). Knee adduction moments are not increased in obese knee osteoarthritis patients during stair negotiation. Gait Posture..

[59] Walter JP, D’Lima DD, Colwell CW, Fregly BJ (2010). Decreased knee adduction moment does not guarantee decreased medial contact force during gait. J. Orthop. Res..

[60] Winby CR, Lloyd DG, Besier TF, Kirk TB (2009). Muscle and external load contribution to knee joint contact loads during normal gait. J. Biomech..

